# Combined Video Laryngoscope and Fiberoptic Nasal Intubation

**DOI:** 10.7759/cureus.19482

**Published:** 2021-11-11

**Authors:** Stacey M Nedrud, Douglas G Baasch, John D Cabral, Daniel S McEwen, Jayanth Dasika

**Affiliations:** 1 Oral and Maxillofacial Surgery, University of Florida, Jacksonville, USA; 2 Oral and Maxillofacial Surgery, Kitsap Oral, Maxillofacial & Dental Implant Surgery, Spokane, USA; 3 Anesthesiology, University of Florida, Jacksonville, USA

**Keywords:** hybrid intubation, difficult airway, fiberoptic intubation, video laryngoscope, nasal intubation

## Abstract

Techniques for facilitating nasal intubation and reducing associated airway trauma are well documented in the literature. This case series describes an additional technique that combines the use of the video laryngoscope and fiberoptic bronchoscope for intubation. Rather than first starting with the fiberoptic bronchoscope, an endotracheal tube is passed through the nasopharynx and lined up with the glottis using video laryngoscopy. Subsequently, the fiberoptic bronchoscope is used only to guide the endotracheal tube through the glottis. The two perspectives simultaneously provide enhanced guidance to the operator, which can, in turn, reduce the burden of fiberoptic navigation through blood, secretions, and/or altered airway anatomy. Additionally, our report demonstrates that this procedure can be used as a rescue measure when Magill forceps are unsuccessful.

## Introduction

Nasal intubation is frequently achieved with either direct or video laryngoscopy with or without Magill forceps [1,2]. Trauma can be minimized by the use of softened endotracheal tubes, red rubber catheters, lubrication, topical vasoconstrictors, and dilation of the nares [3-5]. Despite these measures, oral manipulation of the tube can lead to tissue injury and endotracheal tube cuff damage. In addition, the endotracheal tube may not pass through the glottis easily causing additional trauma and a higher incidence of postoperative pharyngitis [6,7]. For known difficult airways, many practitioners utilize a strict nasal fiberoptic approach. Recently, another approach has been described by Abrons et al. using a pediatric bougie via a Seldinger technique [8,9], wherein challenging nasal airways are secured without the need for either a fiberoptic bronchoscope or Magill forceps. The hybrid technique we describe here utilizes both video laryngoscopy and fiberoptic navigation, which can be useful electively or, more likely, as a rescue measure for difficult airways.

## Case presentation

**Figure 1:**
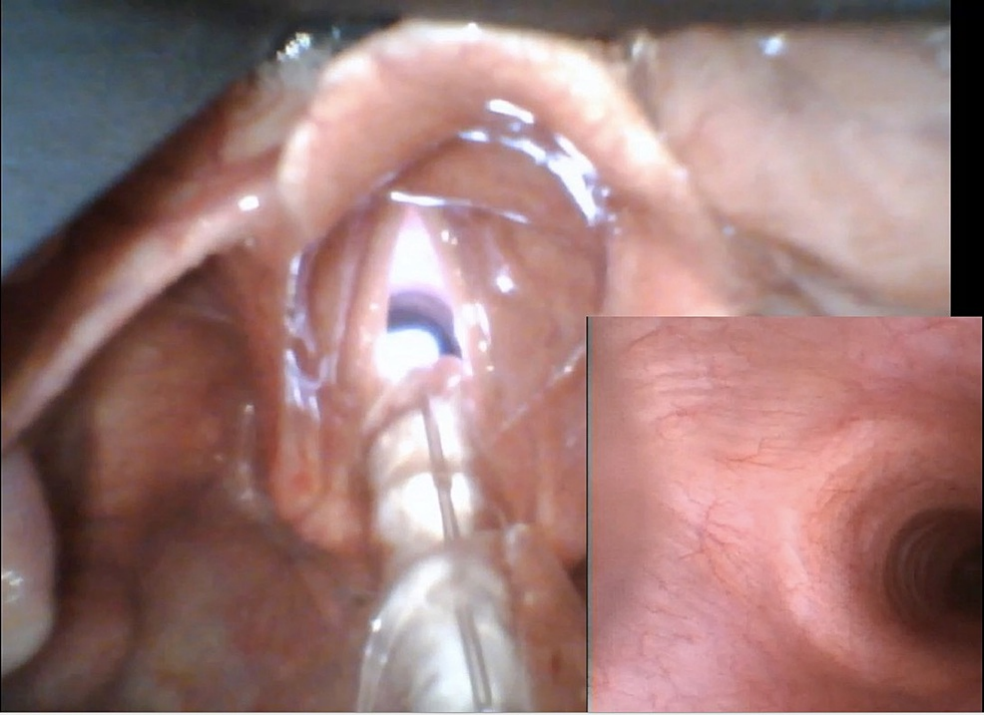
Intubation technique. Picture-in-picture view of intubation using Glidescope® Core™ (Verathon Inc., Bothell, WA, USA). A separate video laryngoscope and fiberoptic bronchoscope can also be used.

In our hybrid technique, after the intravenous induction of general anesthesia and topicalization with lidocaine and oxymetazoline, a nasal Ring-Adair-Elwyn (RAE) endotracheal tube is passed through the favorable nostril and into the nasopharynx. The video laryngoscope is then used to visualize the glottis. Once the tip of the nasal RAE is advanced and visualized near the glottic opening, a fiberoptic bronchoscope is passed through the nasal RAE. One provider is needed to straighten the nasal RAE to avoid damaging the bronchoscope. Both video screens are used simultaneously to guide the fiberoptic bronchoscope and, subsequently, the endotracheal tube through the vocal cords (Figure 1).

We selected three cases to demonstrate the feasibility of this intubation technique. Patients were selected based on a favorable airway examination. In the third case, we demonstrate the use of the technique as a rescue measure. In all three cases, intubation was successful while avoiding trauma to the pharynx. In addition, all endotracheal tubes were passed through the glottis with minimal manipulation or force. There were no reductions in SpO_2_ below 95%. All three cases are discussed below.

Case 1

A 37-year-old male with a history of severe obstructive sleep apnea (OSA) presented for LeFort 1 advancement, bilateral sagittal split osteotomy advancement (i.e., maxillo-mandibular advancement), as well as genioglossus advancement. His airway examination was significant for a Mallampati 1 classification with full mouth opening and neck extension. Due to the surgical requirement for a nasal endotracheal tube, he was planned for intubation via the above-described technique. After preoxygenation, the patient was induced intravenously with lidocaine, fentanyl, propofol, and rocuronium. The intubation proceeded uneventfully and without trauma to the airway, and was successfully completed within five minutes from the time of induction.

Case 2

A 63-year-old male with a history of OSA presented for placement of multiple dental implants, which necessitated nasal intubation for adequate surgical access. His airway examination was significant for a Mallampati 2 classification with full mouth opening and neck extension. After preoxygenation, the patient was induced intravenously with lidocaine, fentanyl, propofol, and rocuronium. Again, the above technique was utilized without complication. Although the endotracheal tube did not line up well with the glottis, navigation of the endotracheal tube into the trachea was successful with the fiberoptic bronchoscope. The patient required one intubation attempt, and the airway was successfully completed within four minutes from the time of induction.

Case 3

A 71-year-old male with a history of squamous cell carcinoma of the oral cavity presented for partial glossectomy and tracheostomy. The airway examination was significant for a Mallampati 3 classification. However, because neck extension and thyromental distance were normal, it was thought that the patient could be intubated nasally via traditional video laryngoscopy and Magill forceps. After preoxygenation, induction proceeded with intravenous lidocaine, fentanyl, propofol, and rocuronium. The endotracheal tube was passed without visible trauma through the right nare. Video laryngoscopy revealed good alignment of the endotracheal tube and the glottis. The tip of the endotracheal tube was grasped orally using Magill forceps and advanced; however, there was significant resistance to advancing the tip beyond the glottis. The Magill forceps was removed and the fiberoptic bronchoscope was introduced down the endotracheal tube through the glottis. Subsequently, the endotracheal tube was advanced easily into the trachea at this point. The patient was intubated within four minutes from the time of induction.

## Discussion

Nasal intubations may be challenging if there is nasopharyngeal bleeding, altered airway anatomy, or difficulty in advancing the endotracheal tube through the glottis. The use of Magill forceps is not always successful and is associated with endotracheal tube cuff damage and postoperative pharyngitis [6,7]. In addition, the video laryngoscope blade often does not leave enough room in the oropharynx for Magill forceps. In case of trauma, blood and secretions may inhibit navigation with just a fiberoptic bronchoscope despite preventative measures [4,5].

Our combined approach is similar to the Seldinger technique demonstrated by Abrons et al. using a pediatric bougie as a guide for the endotracheal tube [8,9]. The main difference is that the nasal RAE is placed using video laryngoscopy just superior to the glottic opening, and the fiberoptic bronchoscope is then passed through the endotracheal tube in lieu of a bougie or Magill forceps. Importantly, by first lining up the endotracheal tube with the glottis, navigation with the fiberoptic bronchoscope is simplified. This can be compared to intubating through a laryngeal mask airway [10], although in this case the endotracheal tube is used as the conduit. Moreover, if Magill forceps are unsuccessful, as demonstrated in our third case, the fiberoptic bronchoscope can be used as a rescue measure. This avoids having to remove the nasal RAE from the oropharynx prior to reattempting intubation. We hypothesize that this technique may result in a similar reduction of trauma, particularly postoperative pharyngitis [7] when compared to the traditional approach using Magill forceps, although this would require further investigation.

While the approach can be used electively, in reality, most facilities have a limited supply of difficult airway equipment. In addition, at least three operators are necessary for intubation; one to manage the video laryngoscope, another to straighten to the nasal RAE, and a third to drive the fiberoptic bronchoscope. Therefore, its most valuable use may be in known difficult airways or as a rescue approach in cases where it is difficult to pass an endotracheal tube with either Magill forceps or a strict fiberoptic approach.

## Conclusions

Nasal intubations using traditional methods may fail, prompting the need for advanced airway techniques. The combined use of a video laryngoscope and fiberoptic bronchoscope may be useful in these situations by providing two different visual planes as the operator approaches the glottic opening. The video laryngoscope provides a more panoramic viewpoint, while the fiberoptic bronchoscope provides a close view of the glottic folds. The cases described here demonstrate the feasibility of this approach and its application as a rescue measure. It is also possible to complete intubation even if the tip of the endotracheal tube does not line up with the glottis. There may also be a reduction in trauma to the airway, particularly concerning postoperative pharyngitis.
